# Telemedicine as support for primary care referrals to neurologists: decision-making between different specialists when guiding the case over the phone

**DOI:** 10.1590/0004-282X-ANP-2020-0137

**Published:** 2021-05-08

**Authors:** Carlos Eduardo MANTESE, Emanuelle Roberta da Silva AQUINO, Mariana Duque FIGUEIRA, Livia RODRIGUES, Josue BASSO, Priscila RAUPP DA ROSA

**Affiliations:** 1 Hospital Sírio-Libanês, São Paulo SP, Brazil. Hospital Sírio-Libanês Hospital Sírio-Libanês São Paulo SP Brazil; 2 Universidade Nove de Julho, Faculdade de Medicina São Paulo SP, Brazil. Universidade Nove de Julho Universidade Nove de Julho Faculdade de Medicina São Paulo SP Brazil

**Keywords:** Telemedicine, Health Services Accessibility, Health Equity, Primary Health Care, Neurology, Telemedicina, Acesso aos Serviços de Saúde, Equidade em Saúde, Atenção Primária à Saúde, Neurologia

## Abstract

**Background:**

Brazil’s public health system, the largest in the world, faces great challenges in establishing access to specialized care for all Brazilians. “Regula Mais Brasil” is a structuring project from the Ministry of Health that aims to optimize the referral process for specialized care throughout Brazil and uses telemedicine to support primary care physicians. The high demand for support and referrals in the neurology specialty raises questions about what the difficulties and doubts in managing these patients are and whether the training of the coordinating physician influences the orientation and outcome of referral.

**Methods:**

In a retrospective analysis on a database, all teleconsultations in the neurology specialty were evaluated. The diagnoses were categorized using the ICD-10 and according to the specialty of the coordinating teleconsultant for multiple comparisons.

**Results:**

In total, 1687 teleconsultations were conducted between January 10 and December 2, 2019, in the cities of Belo Horizonte and Porto Alegre and in the Federal District. The most frequent area of doubt for doctors was about epilepsy. After discussion via telemedicine, 25% of the referrals were avoided and the specialty of the teleconsultant physician did not impact the decision made: 72.3, 72.6 and 66.5%, in relation to approval by neurologists, family doctors and other experts, respectively.

**Conclusion:**

Increasing access to specialists, not only for patients but also for doctors, helps in achieving early resolution of issues of greater difficulty for primary healthcare doctors, thus resulting in lower numbers of referrals.

## RESUMO

**Introdução:**

O maior sistema público de saúde do mundo tem um grande desafio para estabelecer acesso à atenção especializada a todos os brasileiros. O Regula Mais Brasil é um projeto estruturante do Ministério da Saúde que visa otimizar o processo de referenciamento para atenção especializada no território brasileiro e utiliza a telemedicina para suporte de médicos da atenção primária. A elevada procura de apoio e encaminhamento na especialidade de neurologia desperta o questionamento sobre quais são as dificuldades e dúvidas no manejo desses pacientes e se a formação do médico regulador influencia a orientação e o desfecho do encaminhamento.

**Métodos:**

Em uma análise retrospectiva do banco de dados, foram avaliadas todas as teleconsultorias da especialidade de neurologia, os diagnósticos foram categorizados pelo CID-10 e por especialidade do teleconsultor regulador para comparações múltiplas.

**Resultados:**

No total, 1.687 teleconsultorias foram realizadas de 10 de janeiro a 2 de dezembro de 2019, nas cidades de Belo Horizonte, Porto Alegre e Distrito Federal. A área de dúvida mais frequente dos médicos era sobre epilepsia. Após a discussão por telemedicina, 25% dos encaminhamentos foram evitados e a especialidade do médico teleconsultor não impactou na decisão tomada (72,3, 72,6 e 66,5%) em relação à aprovação por neurologistas, médico de família e outros especialistas, respectivamente.

**Conclusão:**

Aumentar o acesso não apenas de pacientes, mas dos médicos aos especialistas contribui para que os temas de maior dificuldade para os médicos da Atenção Primária à Saúde sejam resolvidos precocemente, resultando em um menor número de encaminhamentos.

## INTRODUCTION

Brazil’s national health system, the *Sistema Único de Saúde* (SUS) has undergone major changes over the last 20 years: a structured and coordinated healthcare system has been created and primary care has been defined as the main setting of the healthcare system. Despite these changes, the population complains about difficult access to medical care and fragmentation of its service network, with important gaps between primary and specialized healthcare. Many referrals of patients to specialized care are made without adequate information, without definition of priority level and without counter-referral. Consequently, referral processes frequently result in lack of continuity of care and delays in health assistance^1^^,^^2^^,^^3^^,^^4^.

There is a pent-up high demand for consultations within secondary and tertiary care. Especially in neurology, there are long waiting lists for consultations. In the city of Porto Alegre, in the south of Brazil, the average waiting time for a neurology consultation was 342 days in December 2018^5^. Nonetheless, the complexity of the cases does not seem to justify the increasing numbers of referrals to neurologist. Studies in Brazil have reported that the main complaints in neurological clinics are headache, secondary prevention of stroke, epilepsy follow-up^6^ and diagnoses of dementia^7^, depending on the cohort.

Approximately 80% of cases can be diagnosed and resolved within primary care by a good clinical doctor with adequate training and support. Through this, referral of serious or surgical cases can be prioritized, considering that delays in diagnosing and treating neurological conditions can worsen patient care and quality of life^8^^,^^9^^,^^10^. Telemedicine may be a solution for supporting doctors in remote areas in a continental country like Brazil who have different levels of training due to the absence of any policy of continuing medical education. This could help primary care physicians (PCPs) to manage some neurological conditions in the primary care setting^11^, as already reported in Canada by Bradi et al., where direct communication between PCPs and specialists through the eConsult system avoids 30% of referrals^12^.

In a pragmatic effort to solve the related problems of referrals from primary healthcare (PHC) to different specialists, the Brazilian Ministry of Health (MH) launched the project “Regula Mais Brasil” in a public-private partnership with Hospital Sírio-Libanês, a major private hospital in São Paulo. This was inspired by the successful experience of the pilot telehealth study TelessaúdeRS-UFRGS in the south of Brazil^13^. The “Regula Mais Brasil” goal is to qualify the management of patients with frequent problems within primary health care and to reduce the waiting times for specialized consultations through an e-referral system and telephone-based support in different Brazilian regions.

The objectives of this article were to describe the most frequent doubts presented by PCPs during teleconsultations, to compare decision-making between neurologists or other specialists when guiding the conduct of cases over the phone and to evaluate how a teleconsulting service between doctors can reduce the numbers of unnecessary referrals to secondary and tertiary care.

## METHODS

In this partial analysis on the “Regula Mais Brasil” project, we report on 1687 neurology teleconsultations that were carried out between January 10, 2019, and December 2, 2019. As part of the institutional development support program for SUS, a federal law of 2009 determines that hospitals certified for excellence will receive tax exemptions to subsidize projects. The thematic areas and purposes of these projects are defined by the Ministry of Health, along with the population or cities to be benefited. It is the responsibility of hospitals of excellence to deliver the scientific production, infrastructure and payments of the professionals that are needed to carry out these projects.

### Project infrastructure and logistics

The technological infrastructure was formed by three call centers located in São Paulo, Porto Alegre and Brasília. Information on this has been published in another study^14^.

**Figure 1.**
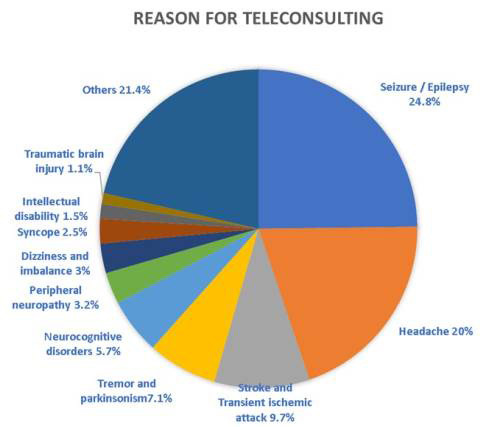
Causes of teleconsultation according to group of disorders.

A free call phone line is open Mondays through Fridays, from 8:00 a.m. to 5:30 p.m., to PCPs in two cities (Porto Alegre, capital and largest city of Rio Grande do Sul, and Belo Horizonte, capital and largest city of Minas Gerais), one state (Amazonas) and the Brazilian Federal District (Figure 1). The participating locations were determined by the Ministry of Health and obeyed predefined criteria: intervention by local governments with the Ministry of Health requesting support, length of the queue, waiting time and number of judicial procedures that had been filed, demanding consultations with specialists. Neurology assistance by means of teleconsultation is available for Porto Alegre, Belo Horizonte and the Federal District, totaling a population of 6 million people involved on this study. Through teleconsultations, not only neurologists, but also family physicians and other specialists answer the PCP questions in real time, using evidence-based-medicine protocols and guidelines, and taking local conditions into consideration. Referral decisions are based on the referral protocols for consultations with specialists, as published by the Health Department of Rio Grande do Sul, and approved by the Ministry of Health for use in the project. The document considered nine pathological conditions that would be sensitive to PHC and guided referrals for emergencies in severe cases or in the presence of warning signs. The final decision about referral always belonged to the PCP and the legal accountability was shared between the physicians.

Through this service, PCPs have the opportunity to discuss cases and decide, with the specialist support, whether referral is required. Furthermore, they can discuss the management of clinical cases in the primary care setting, whether the patient has been referred to a specialist or not. No contact between the patient and teleconsultant occurs during the e-Consult process. This is a teleconsultation service that does not extend to patients. It is important to note that for a case to be referred, the PCP needs to submit the case description via the online referral platform for reading and evaluation by the teleconsulting doctors of the “Regula Mais Brasil” project. To make the call, prior referral via the platform is not mandatory. Thus, many PCPs prefer to first discuss the case over the phone and then submit it on the platform.

Patients are not required to provide their informed consent because the research ethics committee has taken the view that the intervention that the project is implementing is a modification in the patient management process that does not harm the patient’s autonomy. The “Regula Mais Brasil” project has been approved by the ethics committee of Hospital Sírio-Libanês.

A cost-effectiveness study is being carried out to inform the Brazilian government whether the telemedicine strategy is associated with improvement of the patient management process.

### Statistical analysis

A qualitative evaluation was done by one of the neurologist teams using Business intelligence tools. Teleconsultations were grouped according to decision, place, reason for referral and expert who conducted the discussion. A qualitative analysis was performed by an auditing physician, to identify the type of orientation and the need for a second opinion between teleconsultants.

A descriptive analysis was conducted with the aim of presenting the characteristics of the patients who were referred and the referral requests. The questions were categorized according to topic and type and were analyzed quantitatively. Categorical variables were expressed as the absolute frequency (n) and percentage (%). Quantitative variables were described as the mean and standard deviation. Associations between categorical variables were ascertained using the chi-square and Fisher's exact tests. Analyses with p<0.05 were considered statistically significant.

## RESULTS

A total of 1687 teleconsultations relating to neurology, with data available, were conducted between January 10 and December 2, 2019, from the cities of Belo Horizonte and Porto Alegre, and from the Federal District. During this period, the project received 10,780 calls relating to all 12 specialties served. The average length of time per call was 6.8 minutes. The city of Belo Horizonte had the highest number of calls, totaling 1,388 cases discussed over the period. The most common international disease codes (ICD-10) were G40 (epilepsy and idiopathic epileptic syndromes defined by their (focal) (partial) location with focal onset seizures), R51 (headache) and I64 (stroke, not specified as hemorrhagic or ischemic), respectively 23.7, 17.0 and 8.2% (Figure 1). The patients’ mean age was 49.5 years and 58.6% were women.

Most of the cases (54.2%) were discussed with a neurologist, while 35.1% were discussed by a PHC and 10.7% by physicians in other specialties, guided by protocol decisions. The service is available 47.5 h per week and neurologists cover 73% of the service period (Table 1).

**Table 1.** Epidemiological and demographic characteristics.

Sociodemographic characteristics	
Age, mean (SD)	49.5 (20.8)
Place (n, %)	
Belo Horizonte	1,388 (82.3)
Porto Alegre	151 (8.9)
Federal District	148 (8.8)

An overall analysis on the sample showed that 25% of the referrals were avoided using the teleconsultations and that there were no differences in decision made, based on the teleconsultant physician’s specialty (approval indexes of 72.3, 72.6 and 66.5% for neurologists, family physicians and other specialists, respectively) among cases referred for teleconsultation.

Comparison of neurologists versus family doctors or physicians certified in other specialties showed that there were no statistical differences between the numbers of patients approved for specialist consultation, referred to an emergency service or returned for PHC resolution. However, in comparison with family physicians, teleconsultants from focal medical specialties other than neurology guided more case management at PHC units (33.5 vs. 25%; p=0.042), through returning referrals to PHC, while family physicians referred patients more often to an emergency service than did physicians in other specialties (2.4 vs. 0%; p=0.042).

Out of the total, 906 cases were discussed by PCPs in an attempt to accelerate referrals that had already been completed on the online platform but had not yet been attended at the secondary center. Of these, 72.7% were approved for consultations with neurologists. In the meantime, 663 phone contacts were made before the PCPs send the referrals, i.e. these cases was not submitted to the online platform. The objective was to ask for orientation in making decisions about diagnoses and treatments. Out of these, 60.1% were cases that could not be resolved at the PHC unit, and referral was approved by the teleconsultant. Placing the referral on the referral platform before the discussion seems to influence the teleconsultation outcome, given that the number of approvals is greater than it is in cases with discussion without previous referral (72.7 vs. 60.1%; p<0.000). The decision rates according to the teleconsultant's specialty are shown in Table 2.

**Table 2.** Decision rate according to the teleconsultant's specialty.

Cases already referred for consultation with a neurologist			
Teleconsultant’s specialty (total=906)	Referral to a neurologist, n (%)	Recommendation of treatment and follow-up at PHC, n (%)	
Neurologist (452)	353 (78.1)	99 (21.9)	
Family Medicine (340)	254 (74.7)	86 (25.3)	
Other specialties (114)	85 (74.6)	29 (25.4)	
**Cases with shared decision-making before the referral**			
**Teleconsultant’s specialty (total=663)**	**Referral to a neurologist, n (%)**	**Recommendation of treatment at PHC, n (%)**	**Referral to emergency service, n (%)**
Neurologist (401)	254 (63.3)	130 (32.4)	17 (4.2)
Family Medicine (214)	145 (67.8)	65 (30.4)	4 (1.9)
Other specialties (48)	33 (68.8)	10 (20.8)	5 (10.4)

Complete data were available for a total of 1,569 cases.

A detailed analysis of the teleconsulting electronic records showed that neurologist teleconsultants recommended treatment or diagnostic tests more often than did family physicians or other focal specialist teleconsultants (26.1, 17.3 and 28.9%; p=0.000, Pearson chi-square).

Out of the 1687 cases discussed, the diagnosis or treatment approach in 394 cases (23.4%) was altered through discussion of the case, which gave rise to suggestions for the diagnosis or treatment (Table 3).

**Table 3.** Teleconsultants’ recommendations of treatment or diagnostic tests, stratified according to teleconsultant specialties.

Specialty (total=1,687)	Cases with diagnostic or therapeutic orientation, n (%)
Neurologist (919)	240 (26.1)
Family Medicine (588)	102 (17.3)
Other specialties (180)	52 (28.9)

Out of all the cases seen, 23.4% received some type of guidance regarding treatment and diagnosis. PCPs tended to advise less frequently than did other specialists, in a significant way (p<0.000).

## DISCUSSION

This is one of the first published papers about telehealth in neurology in Brazil. In a scenario of scarcity of data on the attendance of certain pathological conditions within PHC, this study makes a contribution through bringing to light the profile of the frequent doubts of PHC doctors regarding the neurology specialty. It confirms that telemedicine is a tool that may add to the care provided for patients. In this project, the referral process based on teleconsultation avoided almost 30% of specialist referrals, which corroborates the findings from studies conducted in other countries with different public healthcare setups^12^^,^^15^. In one of these, in Canada, eConsult was able to avoid 34% of referrals, and the PCPs received information for a new course of action^12^.

Referral systems are organizational structures that play an important role in improving access to care. They bridge the gaps between levels of care, prioritize cases of greater severity and avoid unnecessary referrals^16^^,^^17^^,^^18^^,^^19^. Specialists are limited in number and have irregular geographical distribution. A teleconference between a PCP and a neurologist may anticipate the patient’s treatment and even avoid long-distance travel for this patient. Improvement in technology has allowed some problems to be managed more easily, and telephone support for primary care can make up for some professional shortages in poorer regions. Nonetheless, even if this increases the ability of PHC to resolve cases, we believe that investments in and policies to promote continuing medical education for PCPs is crucial for improving healthcare in Brazil.

Future results from the “Regula Mais Brasil” project may provide support for defining public policies aimed towards structuring complex regional telemedicine coordination and improving the population's access to specialists. A project that was scheduled for completion in December 2020 has already evaluated 342,000 cases and aims to reach 500,000 people in five locations.

Neurological conditions are highly prevalent among patients who seek healthcare and PCPs routinely diagnose and manage many common neurological diseases by taking a holistic view in which the patient’s context is understood^20^. Opportunities to apply teleneurology beyond acute ischemic stroke have encouraged formation of a network to enable patients to have an early diagnosis and adequate treatment, thereby increasing the scope of neurologists’ assistance when approached from PHC via telemedicine.

The heterogeneity of neurologist distribution within Brazil is an important factor to be considered. Our study involved regions with a higher average number of neurologists. Overall, Brazil has an average of 2.46 neurologists per 100,000 inhabitants^21^, i.e., greater than the average of 0.7 for all the Americas^22^. The average for the Federal District is 6.18; Porto Alegre (state of Rio Grande do Sul) 3.66; and Belo Horizonte (state of Minas Gerais) 2.38^21^. However, some locations in Brazil, like most of the states in the northern region, have less than 1.0 neurologist per 100,000 inhabitants (the state of Amazonas has 1.16), and the geographical distribution of these specialists within states is unequal^21^. It is also important to realize that these rates include all the specialists registered in Brazil and do not represent the rate of specialists available for PHC referrals within the public secondary and tertiary networks. In addition, the number of specialist consultations available to patients who depend exclusively on the public healthcare system is not always proportional to these rates. These considerations lead us to infer that in the north of Brazil, use of telemedicine to support healthcare professionals may be more effective than in the cities evaluated in this paper.

As a non-randomized study, this investigation was not appropriate for testing the agreement between assessments made by neurologists versus those made by physicians in other specialties during discussions on cases. However, it could be seen that there was no difference in outcome between neurologists and physicians in other specialties. These preliminary findings show the feasibility of providing training and comprehensive protocols for physicians with generalist training, to enable them to screen for neurological disease that are sensitive to PHC, such as headache and secondary prevention post-stroke. Nevertheless, neurologists seemed to contribute more information about treatment during teleconsultations, which leads us to speculate whether this might be related to the reliability of receiving direct recommendations from specialists.

Our focus in this paper was to show that referrals can be avoided through telehealth support. One limitation of our study was that we did not follow the cases that we have discussed to ascertain their outcomes. Therefore, we do not know whether patients had searched for another way to consult a neurologist, by using the supplementary healthcare system (private system) or finding a neurologist in the public healthcare system without going through the referral system. However, we believe that this is not necessarily a bias, because the PCPs could rediscuss the cases whenever they needed to and had the central role in making referral decisions.

In the emergency context, use of telemedicine in neurology is widespread, with expansion of telestroke coverage over the last decade in different countries. However, there are also opportunities for use of telemedicine in managing neurological disorders^23^. These include replacing or complementing in-office evaluations, decreasing the time between follow-up visits, reducing the disparities in access to healthcare and promoting education and training through interactions between PCPs and tertiary referral centers.

Telehealth support can help PCPs avoid 29% of referrals. This could be used in locations where there are shortages of neurologists and could help to reduce care costs. In Brazil, the country with the fifth largest population in the world, with continental dimensions, regional social and cultural differences and inequity in medical service availability, telehealth could be used to make up for these differences, thereby improving medical outcomes and patient access to healthcare at the appropriate level of complexity.
